# Ligelizumab improves angioedema, disease severity and quality-of-life in patients with chronic spontaneous urticaria^☆^

**DOI:** 10.1016/j.waojou.2022.100716

**Published:** 2022-11-15

**Authors:** Martin Metz, Jonathan A. Bernstein, Ana M. Giménez-Arnau, Michihiro Hide, Marcus Maurer, Karl Sitz, Weily Soong, Gordon Sussman, Eva Hua, Avantika Barve, Nathalie Barbier, Maria-Magdalena Balp, Thomas Severin

**Affiliations:** a Institute of Allergology, Charité – Universitätsmedizin Berlin, Corporate Member of Freie Universität Berlin and Humboldt-Universität zu Berlin, Berlin, Germany; b Fraunhofer Institute for Translational Medicine and Pharmacology ITMP, Allergology and Immunology, Berlin, Germany; c University of Cincinnati College of Medicine and Bernstein Clinical Research Center, Cincinnati, OH, United States; d Dermatology Department, Hospital Del Mar-Parc de Salut Mar, IMIM Universitat Autònoma and Universitat Pompeu Fabra, Barcelona, Spain; e Department of Dermatology, Hiroshima University, Hiroshima, Japan; f Department of Dermatology, Hiroshima City Hospital, Hiroshima, Japan; g Little Rock Allergy and Asthma Clinic, Little Rock, AR, United States; h AllerVie Health-Alabama Allergy and Asthma Center, Clinical Research Center of Alabama, Birmingham, AL, USA; i Division of Allergy and Clinical Immunology, University of Toronto, Canada; j Shanghai Novartis Trading Ltd., Shanghai, China; k Novartis Pharmaceuticals Corporation, East Hanover, NJ, United States; l Novartis Pharma AG, Basel, Switzerland

**Keywords:** Angioedema, Chronic spontaneous urticaria, Dermatology life quality index, IgE, Ligelizumab

## Abstract

**Background:**

Disease burden is particularly high in Chronic Spontaneous Urticaria (CSU) patients with angioedema, and patients whose signs and symptoms are inadequately controlled by H_1_-antihistamines need new treatment options. Here we report an exploratory analysis, from the ligelizumab Phase 2b study, investigating angioedema occurrence in patients with CSU and describe the changes in angioedema following treatment with ligelizumab, omalizumab, or placebo.

**Methods:**

Data from the ligelizumab Phase 2b core (ligelizumab 72 mg, 240 mg, omalizumab 300 mg and placebo) and extension study (ligelizumab 240 mg) were used. Changes in Weekly Angioedema Activity Score (AAS7), the Dermatology Life Quality Index (DLQI), and Weekly Urticaria Activity Score (UAS7) among each time point were analyzed for each treatment arm.

**Results:**

From a total of 297 patients analyzed, 165 (55.6%) reported angioedema occurrence at baseline, with mean AAS7 ranging 30.6—42.2 across treatment arms. At Week 12 of the core study 87.5%, 84.6%, 75.0%, and 61.0% of patients were angioedema free for ligelizumab 72 mg, 240 mg, omalizumab 300 mg, and placebo arms, respectively. In CSU patients with angioedema at baseline, the largest change from baseline in AAS7 score was observed with ligelizumab 72 mg (−31.9) at week 16 in the core study. Patients with angioedema had a higher mean DLQI at baseline (14.9—16.1) vs. patients without angioedema (10.6—12.0). In patients with angioedema, low AAS7 was significantly associated with complete response on UAS7 (UAS7 = 0) and complete normalization of DLQI (DLQI 0—1).

**Conclusion:**

In the Phase 2b study, ligelizumab effectively reduced angioedema and urticaria symptoms, and improve health related quality of life in patients with moderate-to-severe CSU.

**Clinicaltrails.gov NCT number:**

NCT02477332; NCT02649218.

## Introduction

Studies have indicated that chronic urticaria has a point prevalence of 0.1—1.4%, depending on the region,^1^ two thirds of which are chronic spontaneous urticaria (CSU).^2^ Approximately 40% of patients with CSU experience angioedema,^3^ which is characterized by a sudden pronounced erythematous or painful swelling of the lower dermis and subcutis or mucus membranes, occurring mostly on the face and inside the mouth, but also elsewhere on the body.^4^^,^^5^ A real-world study among inadequately controlled CSU patients revealed that angioedema is under-reported in medical charts compared to patient reports.^6^

Disease burden of CSU is particularly high in patients with angioedema.^2^ Often, patients who experience CSU with concurrent hives and angioedema experience a longer duration of disease compared with patients who only have hives.^7^^,^^8^ Although not life-threatening, angioedema can be disfiguring and painful. Some patients fear that swelling episodes will lead to breathing difficulties, which can restrict daily activities.^9^^,^^10^ Angioedema associated with CSU is debilitating for affected patients, sometimes leading to unnecessary hospitalizations, and challenging for their treating physicians.^11^ Angioedema has a significant additional impact on dermatology related quality of life (QoL), compared to patients without angioedema.^6^^,^^12^ Patients with CSU who also experience angioedema can suffer a poorer health related QoL (HRQoL) compared to those without angioedema.^3^^,^^13^

The global guidelines for treatment of CSU recommend the use of omalizumab, the only licensed anti-immunoglobulin E (IgE) monoclonal antibody for clinical use, as an add-on therapy to H_1_-antihistamines for the treatment of CSU.^4^ Despite ongoing treatment with current standard of care, complete control of CSU and angioedema remains hard to achieve in some patients and additional treatment options are needed.^14^, ^15^, ^16^ Ligelizumab is a next generation, high affinity, 97% humanized, monoclonal anti-IgE antibody which results in rapid, strong, and sustained urticaria control,^17^, ^18^, ^19^, ^20^, ^21^, ^22^ as demonstrated in the Phase 2b core study (ClinicalTrials.gov number: NCT02477332)^18^ followed by a one-year Phase 2b extension study (ClinicalTrials.gov number: NCT02649218).^23^ The ligelizumab clinical Phase 3 studies (NCT03580369 and NCT03580356) recruited more than 2000 CSU patients with or without angioedema, across 48 countries worldwide, and met their primary endpoints of superiority for ligelizumab vs. placebo at Week 12, but not vs. omalizumab. Here, we report the Phase 2b core and extension exploratory analysis data investigating the angioedema occurrence in patients with CSU. We describe the changes in Angioedema Activity Score (AAS) following treatment with ligelizumab, omalizumab, or placebo. The assessment of disease activity in affected patients is important to guide treatment decisions, and patient reported outcome (PRO) data are essential in diagnosing and managing CSU. Therefore, to evaluate the efficacy of ligelizumab in patients with CSU, with and without angioedema, the Dermatology Life Quality Index (DLQI) and Urticaria Activity Score (UAS) were also measured.

## Methods

### Study design

The study design has been previously reported in detail.^18^ Briefly, in the double-blind, active- and placebo-controlled Phase 2b study, adult patients with moderate-to-severe CSU (defined by Weekly Urticaria Activity scores [UAS7] ≥16 on a scale from 0 to 42, with higher scores indicating greater disease activity) were randomized to receive ligelizumab 240 mg, 72 mg, 24 mg, omalizumab 300 mg or placebo every 4 weeks (q4w), or a single dose of ligelizumab 120 mg at randomization followed by placebo q4w (2:2:2:1:1:1). All patients received H_1_ antihistamine background therapy. For the analysis presented in this manuscript, patients from the ligelizumab 24 mg (below the therapeutic dose-response range) and 120 mg single dose (purposed for pharmacokinetic-pharmacodynamic assessments) treatment arms of the core study are not included. However, for any analysis regarding the extension study, all patients that entered the extension study are considered (Supplementary Figure 1). The institutional review board at each participating center approved the study protocol. Patients provided written informed consent to participate in the study. The study was conducted according to the ethical principles of the Declaration of Helsinki.

### Participants

Adult patients (≥18 to ≤75) diagnosed with refractory CSU who remained symptomatic despite treatment with H_1_-anthistamines at approved or increased doses, alone or in combination with H_2_-anthistamines and/or a leukotriene receptor antagonist, were eligible to enter the core study. The inclusion and exclusion criteria, and baseline demographic and clinical characteristics of the patients, have previously been described in detail.^18^ The key inclusion criteria for the extension study were patients who remained in the follow-up period of the core study for at least 12 weeks and had active disease (UAS7≥12) (Supplementary Figure S1).

### Exploratory endpoints and statistical analysis

The proportion of patients with CSU with and without angioedema was recorded at baseline, and throughout the Phase 2b core and extension studies. The baseline characteristics of the patient population with and without angioedema are described with summary statistics. Angioedema occurrence and severity were recorded with the AAS, a specific PRO that measures disease activity in patients with recurrent angioedema.^9^ Episodes of angioedema including duration, severity, and impact on daily functioning and appearance were assessed. Angioedema occurrence is reported with descriptive statistics. Daily scores were summed up as the 7-day AAS (AAS7), with total scores ranging between 0 and 105 (a higher score being worse). A patient was considered as having angioedema at baseline when there was at least 1 day during the week prior to baseline visit with an occurrence of swelling as reported on the AAS7.

The mean change from baseline (CFB) in AAS7 for patients with angioedema at baseline was analyzed throughout the studies using a mixed model analysis with age, IgE at baseline, and baseline AAS7 as covariates for the core study and with CSU duration and extension baseline AAS7 for the extension study. Patients with AAS7 = 0 were defined as angioedema free. A step-wise regression was used to identify potential factors influencing the angioedema outcome (AAS7) at Week 20. The least squares (LS) mean for each treatment group was estimated with a repeated measure mixed model using all the selected factors from the stepwise analysis. The number of weeks with and without angioedema and the rate of angioedema and non-angioedema weeks were also evaluated. Analysis of angioedema status by treatment visit was also conducted. To investigate the relationship between AAS7 and UAS7, 2 repeated measure mixed models, 1 for the core study up to Week 32 and 1 for the extension study were used on AAS7 scores with UAS7 responder status at each visit, and age, sex, and duration of CSU as fixed effect factors with subject as a random effect using a compound symmetry covariance matrix.

The mean CFB-UAS7, were evaluated at baseline, and throughout the treatment period. Complete freedom from signs and symptoms of urticaria was indicated by UAS7 = 0. The DLQI, a 10-item dermatology-specific health-related (HR) QoL measure, was completed by all patients at baseline, and every 4 weeks thereafter. A mixed model comparison analysis was conducted between AAS7 and DLQI categories^24^ (0–1 = no effect, 2–5 = small effect, 6–10 = moderate effect, 11–20 = very large effect, and 21–30 = extremely large effect on patient's life), considering DLQI health status at each visit, visit, age, sex, and duration of CSU as fixed effect factors, and subject as a random effect factor.

The implemented statistical analyses had limitations. These were exploratory post hoc analyses with nominal p-values. No multiplicity adjustments were made, therefore, statistical interpretation on the significance level should be made with caution. To ensure that the cross arm pooled data were not challenged by the confounded treatment effects for these exploratory analyses, not all data from the Phase 2b study were used. Ligelizumab dose below the therapeutic dose-response range, and the single dose arm^18^ were not considered in these analyses. For the analyses on angioedema, the subgroup analyses were only done on patients who had angioedema at baseline. This was done to support the conclusion for CSU patients with concomitant angioedema.

## Results

### Participants

Of the total 382 patients randomized in the core study, 88.5% (338) completed the treatment phase; all baseline demographics and clinical characteristics were balanced across treatment arms.^18^ For the present analysis, data of 297 patients from the ligelizumab 72 mg and 240 mg, omalizumab 300 mg and placebo arms of the core study were considered. From the core study population that entered the treatment-free follow-up period (n = 320), 70.6% (226) of patients were included, after the 12-week washout period (at Week 32), to receive ligelizumab 240 mg in the one-year open-label extension study. Baseline demographics, disease characteristics, patient disposition and safety during the core and extension study have been reported previously.^18^ A summary of patient demographics and disease characteristics of patients considered for this analysis is provided in Table 1.

**Table 1:** Baseline demographics and disease characteristics of patients in the Phase 2b core and extension studies

	Core^a^					Extension
	Ligelizumab 72 mg (N = 84)	Ligelizumab 240 mg (N = 85)	Omalizumab 300 mg (N = 85)	Placebo (N = 43)	Total (N = 297)	Ligelizumab 240 mg (N = 226)
Presence of angioedema^b^ n (%)	43 (51.2)	46 (54.1)	48 (56.5)	28 (65.1)	165 (55.6)	84 (37.2)
Age (years)	44.3 ± 12.4	42.9 ± 10.5	41.8 ± 13.1	45.4 ± 11.2	43.4 ± 11.9	44.5 ± 12.7
Angioedema	46.7 ± 13.4	42.8 ± 11.0	42.3 ± 13.1	47.1 ± 10.7	44.4 ± 12.3	45.4 ± 11.8
No angioedema	41.8 ± 10.8	43.1 ± 10.0	41.2 ± 13.1	42.2 ± 11.8	42.1 ± 11.3	44.0 ± 13.2
Female sex, n (%)	61 (72.6)	67 (78.8)	66 (77.6)	31 (72.1)	225 (75.8)	170 (75.2)
Angioedema	32 (74.4)	41 (89.1)	37 (77.1)	19 (67.9)	129 (78.2)	65 (77.4)
No angioedema	29 (70.7)	26 (66.7)	29 (78.4)	12 (80.0)	96 (72.2)	105 (73.9)
BMI	28.5 ± 7.1	27.9 ± 6.1	28.1 ± 6.4	27.4 ± 6.5	28.1 ± 6.5	28.8 ± 7.2
Angioedema	29.0 ± 6.8	27.9 ± 6.1	28.8 ± 7.2	28.9 ± 7.0	28.6 ± 6.7	30.2 ± 7.3
No angioedema	28.1 ± 7.5	27.9 ± 6.2	27.2 ± 5.3	24.7 ± 4.7	27.4 ± 6.3	27.9 ± 7.1
Duration of CSU (years)	3.9 ± 5.4	4.1 ± 5.6	5.1 ± 7.5	3.6 ± 3.5	4.2 ± 5.9	4.8 ± 6.2
Angioedema	4.9 ± 6.1	5.0 ± 6.5	5.7 ± 8.9	3.2 ± 2.9	4.9 ± 6.8	4.6 ± 6.7
No angioedema	2.7 ± 4.3	3.1 ± 4.1	4.3 ± 5.3	4.4 ± 4.5	3.5 ± 4.6	4.8 ± 5.9
AAS7^c^ score	42.2 ± 25.0	32.8 ± 28.1	30.6 ± 22.8	39.5 ± 24.9	35.7 ± 25.5	30.9 ± 24.8
UAS7						
Angioedema	33.1 ± 6.7	29.8 ± 7.5	30.2 ± 8.5	32.0 ± 6.5	31.2 ± 7.5	29.2 ± 8.2
No angioedema	30.2 ± 7.8	30.9 ± 7.1	28.1 ± 7.0	29.4 ± 7.3	29.7 ± 7.3	27.7 ± 9.6
DLQI						
Angioedema	16.1 ± 8.0	15.2 ± 7.9	15.0 ± 6.9	14.9 ± 5.9	15.4 ± 7.3	15.7 ± 6.8
No angioedema	10.9 ± 6.7	12.0 ± 7.1	11.9 ± 6.6	10.6 ± 5.7	11.5 ± 6.6	12.6 ± 7.3

All data are expressed as mean ± standard deviation or n (%).

AAS7, weekly angioedema activity score; BMI, body mass index; CSU, chronic spontaneous urticaria; DLQI, dermatology life quality index; N, full analysis set; n, number of patients; QoL, quality of life; UAS7, weekly urticaria activity score.

a Only data from four treatment groups from the core study are presented.

b Percentage of patients with angioedema non missing evaluation at baseline are presented.

c AAS7 of patients with angioedema at baseline are presented. All patients had a baseline DLQI score >13 suggesting a severe impact on QoL.

### Angioedema occurrence

**Fig. 1:**
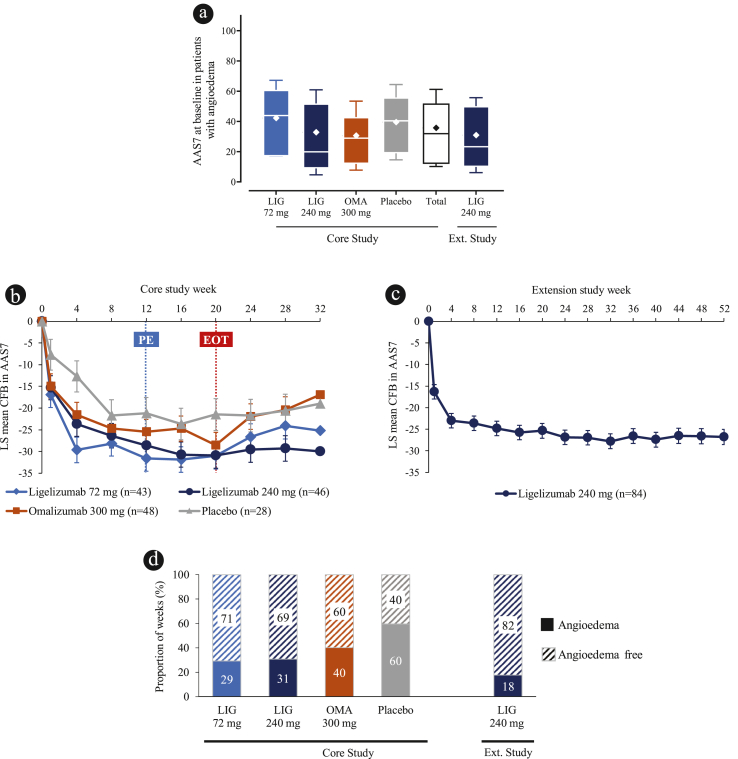
Angioedema status of patients in the ligelizumab Phase 2b studies: (**a)** median (IQ range), mean ± SD of AAS7 at baseline in the core and extension studies, (**b)** LS mean CFB AAS7 in the core Phase 2b study, (**c)** LS mean CFB AAS7 in the extension study, (**d)** mean rate of weeks with or without angioedema in the core and extension studies. Presented at EAACI 2022: https://www.urticariaknowledgecenter.novartis.com/EAACI/presentations/2022/Posters/EAACI_2022_Angioedema_control_Poster.pdf. Box-and-whisker plot shows medians (horizontal line) with Q1-Q3 range (edges of box), error bars represent SD, with a diamond marker within the box depicting mean. LS means from Mixed model of repeated measures (MMRM model): CFB in AAS7 score, considering treatment, visit, baseline total IgE, baseline AAS7 score, age as covariates for core study, and visit and extension baseline AAS7 score as covariates for the extension. Only patients with angioedema at baseline were considered for this analysis. Rate of angioedema is defined as (number of weeks with angioedema in core treatment phase)/(number of completed weeks in core treatment phase). Rate of non-angioedema is defined as: (number of weeks without angioedema in core treatment phase)/(number of completed weeks in core treatment phase). Blue dotted line indicates primary endpoint. Red dotted line indicates the end of the treatment period of the core study. AAS7, weekly angioedema activity score; CFB, change from baseline; Ext., extension; LIG, ligelizumab; LS Mean, least squares mean; n, number of patients; OMA, omalizumab; Q1-Q3, 1st to 3rd interquartile range; SD, standard deviation.

In the core study, a total of 165/297 (55.6%) patients reported to have angioedema at baseline, comprising of 51.2% and 54.1% of patients in the ligelizumab 72 mg and 240 mg groups, respectively, 56.5% for omalizumab 300 mg, and 65.1% for placebo. In the extension study, 84/226 (37.2%) of patients had angioedema at extension study baseline (Table 1). Patients in the present analysis had a numerically higher mean ± SD AAS7 score of 35.7 ± 25.5 at baseline in the Phase 2b core study compared with 30.9 ± 24.8 in the extension study. The Baseline AAS7 among patients with angioedema in the core study was 42.2, 32.8, 30.6 and 39.5 for ligelizumab 72 mg, 240 mg, omalizumab 300 mg and placebo arms, respectively (Table 1 and Fig. 1a).

For all patients in the core study, at Week 12, 87.5%, 84.6%, and 75.0% of patients treated with ligelizumab 72 mg, 240 mg, and omalizumab 300 mg, respectively, were angioedema-free, compared with 61.0% on placebo. This improvement was sustained until the end of the core study at Week 20, and 87.2%, 86.3%, and 77.8% of patients treated with ligelizumab 72 mg, 240 mg and omalizumab 300 mg, respectively, achieved angioedema-free status compared with 65.0% on placebo. Similarly, in the extension study, at Week 12, 88.6% of all patients treated with ligelizumab 240 mg were angioedema-free, which was sustained at Week 20 (92.3% of all patients achieving angioedema-free status). At the end of the extension study treatment period (Week 52), 92.5% of all patients were angioedema-free.

### Changes in AAS7 over time

The LS mean change from baseline (CFB) in AAS7 evaluated for patients with angioedema, by treatment groups is shown in Fig. 1b and **c**. The greatest LS mean ± standard error (SE) CFB-AAS7 was observed for patients treated with ligelizumab 72 mg at Week 16 (−31.9 ± 2.9). At Week 12, CFB-AAS7 for ligelizumab 72 mg, 240 mg, omalizumab and placebo was −31.6 ± 2.9, −28.6 ± 2.8, −25.5 ± 2.9 and −21.2 ± 3.6, respectively. For patients in the extension study, ligelizumab 240 mg showed an LS mean ± SE CFB in AAS7 of −24.8 ± 1.7 at Week 12, −25.4 ± 1.7 at Week 20, and −26.8 ± 1.7 at Week 52. The rate of angioedema free weeks during the treatment period was higher in the ligelizumab and omalizumab arms vs. placebo (Fig. 1d).

### Changes in UAS7

**Fig. 2:**
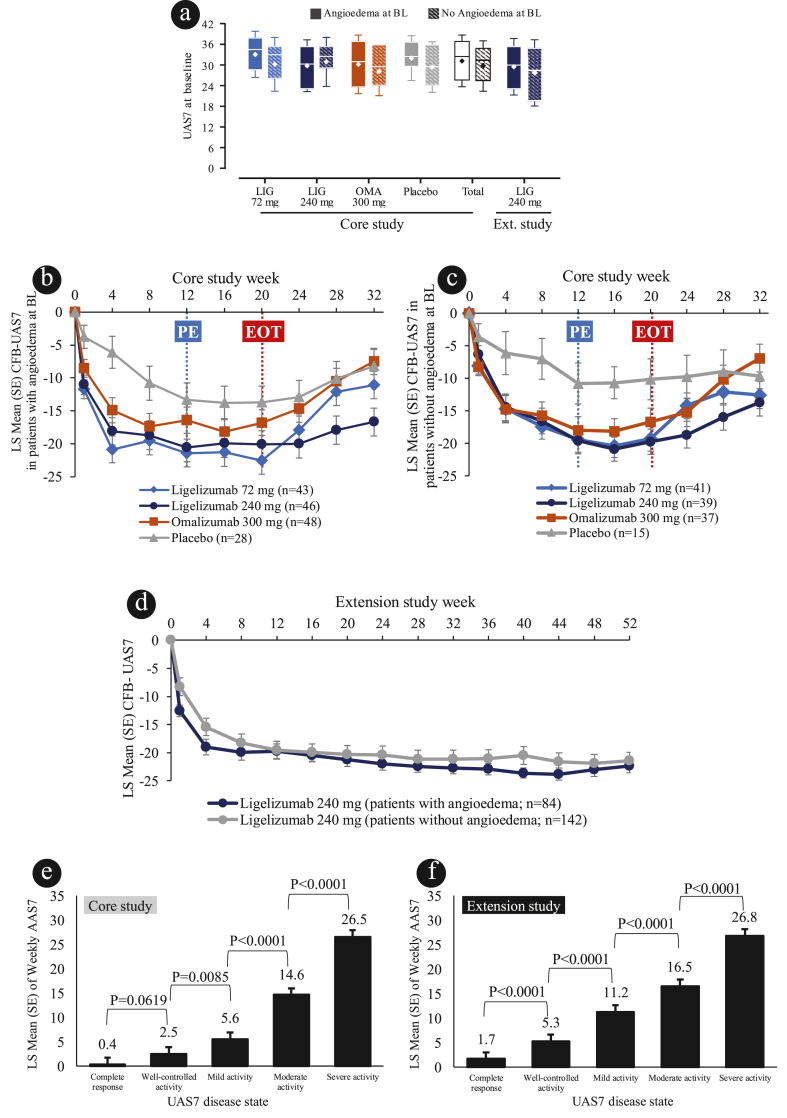
Urticaria activity and co-occurrence of angioedema: (**a)** median (IQ range), mean ± SD UAS7 at baseline in the Phase 2b and studies; mean CFB UAS7 in patients **(b)** with angioedema and **(c)** without angioedema in the core Phase 2b study, and **(d)** with or without angioedema in the extension study; AAS7 by UAS7 responder status among patient with angioedema at baseline in **(e)** core study, and **(f)** extension study. Presented at AAD 2022: https://www.urticariaknowledgecenter.novartis.com/AAD/presentations/2022/AAD_2022_Patients_treated_Poster.pdf. Box-and-whisker plot shows medians (horizontal line) with Q1-Q3 range (edges of box). Error bars represent SD. Diamond marker within the box depicts mean. Blue and red dotted line indicates primary endpoint (Week 12) and end of the treatment period (Week 20), respectively. Patients who remained in the follow-up period for at least 12 weeks and had active disease (UAS7≥12), could enter the extension study from Week 32 onwards for the core study. UAS7 disease activity categories; complete response (UAS7 = 0); well-controlled (UAS7 = 1–6); mild (UAS7 = 7–15); moderate (UAS7 = 16–27); severe (UAS7 = 28–42). AAS7, Weekly Angioedema Activity score; BL, baseline; CFB, change from baseline; Ext., extension; LIG, ligelizumab; LS mean, least squares mean; n, number of patients; OMA, omalizumab; SD, standard deviation; SE, standard error; UAS7, weekly urticaria activity score.

In the core study, mean ± SD UAS7 at baseline for all patients with angioedema (31.2 ± 7.5) was similar to that observed in patients without angioedema at baseline (29.7 ± 7.3). Similarly, in the extension study, mean ± SD UAS7 at baseline for all patients with angioedema (29.2 ± 8.2) was comparable to that observed in patients without angioedema (27.7 ± 9.6; Table 1 and Fig. 2a).

Improvements in mean CFB in UAS7 in patients with angioedema were observed across all treatment groups. For patients with angioedema, the LS mean ± SE CFB-UAS7 at Weeks 4, 12 and 20 were higher (better) for ligelizumab 72 mg (−20.9 ± 2.0, −21.4 ± 2.1 and −22.5 ± 2.1), ligelizumab 240 mg (−18.1 ± 2.1, −20.5 ± 2.1 and −20.1 ± 2.1) and omalizumab 300 mg (−14.9 ± 1.9, −16.4 ± 2.0 and −16.8 ± 2.0) vs. placebo (−6.1 ± 2.4, −13.3 ± 2.6 and −13.8 ± 2.5; Fig. 2b). For patients without angioedema at baseline the CFB-UAS7 was similar between treatment arms, and higher compared with placebo (Fig. 2c). In the extension study, the LS mean ± SE CFB-UAS7 scores, at Weeks 4, 12, 20 and 52 was −19.0 ± 1.4, −19.7 ± 1.3, −21.3 ± 1.2 and −22.4 ± 1.2, respectively, for patients with angioedema. (Fig. 2d).

In the core study, the comparison by UAS7 responder status (pooled data up to Week 32) demonstrated that patients who were free from urticaria signs and symptoms (complete response: UAS7 = 0) had a lower LS mean ± SE AAS7 vs. patients with well-controlled urticaria (0.4 ± 1.4 vs. 2.5 ± 1.4, p = 0.062; Fig. 2e). Similarly, in 52 weeks pooled data from the extension study, the comparison by UAS7 responder status demonstrated that patients achieving UAS7 = 0 had significantly lower LS mean ± SE AAS7 vs. patients with well controlled urticaria (1.7 ± 1.4 vs. 5.3 ± 1.4, p < 0.0001; Fig. 2f).

### Impact of angioedema on DLQI

**Fig. 3:**
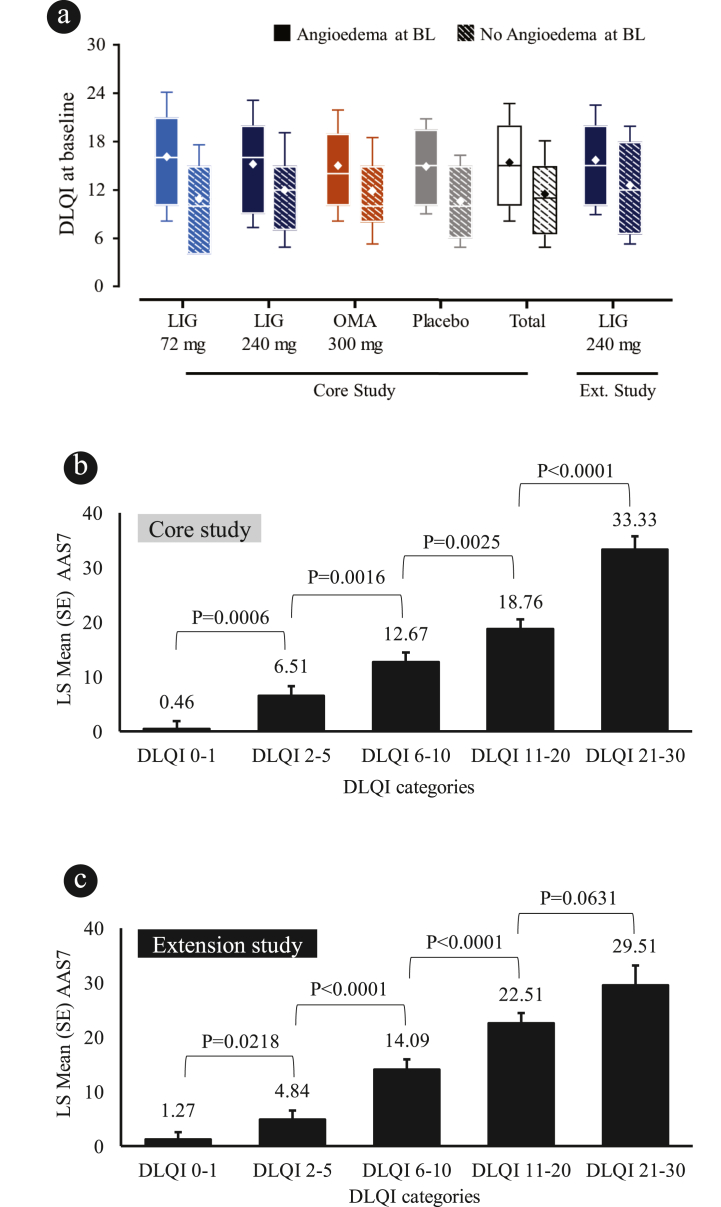
Dermatology-QoL by angioedema status at baseline: **(a)** Median (IQ range), mean ± SD in the Phase 2b core and extension studies. AAS7 comparison by DLQI categories among patient with angioedema at baseline **(b)** in the Phase 2b core study, and **(c)** in the extension study. Presented at EAACI 2022: https://www.urticariaknowledgecenter.novartis.com/EAACI/presentations/2022/Posters/EAACI_2022_Angioedema_control_Poster.pdf. Box-and-whisker plot shows medians (horizontal line) with Q1-Q3 range (edges of box), error bars represent SD, with a diamond marker within the box depicting mean. DLQI categories for effect on QoL (DLQI score 0–1: no effect; 2–5: small effect; 6–10: moderate effect; 11–20: very large effect; and 21–30: extremely large effect). AAS7, weekly angioedema activity score; BL, baseline; DLQI, Dermatology Life Quality Index; Ext., extension; LIG, ligelizumab; OMA, omalizumab; QoL, quality of life; SE, standard error; SD, standard deviation

In the core study, across arms the mean (14.9—16.1) and median (14.0—16.0) baseline DLQI score for patients with angioedema was higher compared to patients without angioedema (mean 10.6—12.0; median 10.0–12.0), indicating a more severe impact on life; Table 1 and Fig. 3a. Similarly, in the extension study, overall, patients with angioedema had a higher mean DLQI score at baseline (15.7) compared with patients without angioedema (12.6; Table 1 and Fig. 3a). In the pooled data up to Week 32 from the core study, the comparison by DLQI categories demonstrated that patients with DLQI 0–1 had a significantly lower LS mean ± SE AAS7 vs. patients with DLQI 2–5 (0.46 ± 1.43 vs. 6.51 ± 1.75, p = 0.0006; Fig. 3b). Similarly, in 52 weeks pooled data from the extension study, the comparison by DLQI categories status demonstrated that patients achieving DLQI 0–1 had significantly lower LS mean ± SE AAS7 vs. patients with DLQI 2–5 (1.27 ± 1.31 vs. 4.84 ± 1.75, p = 0.0218; Fig. 3c). Overall, lower AAS7 were significantly associated with better dermatology-QoL outcomes (Fig. 3 b and c).

### Safety

Safety results for the core study and the extension study have been previously reported. Briefly, all tested doses of ligelizumab showed a safety profile comparable to omalizumab. No adverse events of clinical relevance were observed during the core study,^18^ and no new safety signals were identified in the extension study where all patients received the highest dose (240 mg) of ligelizumab.^23^

## Discussion

Angioedema is a common symptom, albeit fluctuating and unpredictable, that may co-occur with hives and itch in patients with CSU. Even though angioedema is not considered life-threatening in CSU patients, it increases the burden of disease and severely impacts patient's QoL. Chronic spontaneous urticaria patients with angioedema, whose signs and symptoms are inadequately controlled by H_1_-antihistamines, need new treatment options. In the ligelizumab Phase 2b core study, the patient inclusion criteria were not dependent on co-occurrence of angioedema with CSU, but patients were assessed for occurrence of angioedema at baseline as any positive value of AAS7 in the week prior to randomization. This is in contrast to the angioedema focused X-ACT study in which history of angioedema with ≥4 episodes within the last 6 months was the inclusion criteria.^10^ Despite this, more than 56% of patients in this post-hoc analysis of the ligelizumab Phase 2b core study had angioedema at baseline. In addition, the studies enrolled patients with moderate-to-severe disease activity regardless of presence or absence of angioedema at baseline. A greater proportion of patients in the core study had angioedema at baseline compared to the extension study, although the baseline angioedema scores were comparable between the studies. This further highlights the fluctuating nature and unpredictability of angioedema in patients with CSU, especially considering the overall baseline UAS7 was similar between the two studies. However, it was evident from the data that patients with angioedema had a higher impact on dermatology related QoL compared with patients without angioedema.

The reported exploratory analysis showed that CSU patients with angioedema who were treated with ligelizumab experienced significant improvements in AAS7 and UAS7. A large number of patients were angioedema-free by Week 12 of both studies, and patients treated with ligelizumab and omalizumab experienced a greater CFB in AAS7 scores over time compared with placebo. In the core study, all patients with CSU and angioedema that received ligelizumab or omalizumab treatments, achieved improvements in AAS7 and UAS7 that were sustained over time. In agreement with the previously reported ligelizumab dose response relationship in terms of complete response on hives,^18^ the efficacy of ligelizumab on angioedema was similar between the 72 mg and 120 mg doses. Improvements in mean CFB AAS7 scores were observed throughout the treatment phase of the core study and follow-up periods. Similarly, in the extension study, re-treatment with ligelizumab 240 mg was effective in reducing AAS7 for all patients who were included from the core study. The efficacy of re-treatment with ligelizumab 240 mg could be indicative of sustained response on continued therapy, as over 92.5% of patients reached angioedema-free status by the end of the extension study treatment period.

Angioedema can be a debilitating condition and is frustrating for both patients and physicians. In patients presenting with CSU it is estimated that 33–67% of cases will have associated angioedema.^3^^,^^10^ Similarly, in our study,^18^ based on the AAS7 score, over 50% of patients reported having angioedema at baseline. The results of the ASSURE study showed that CSU patients with angioedema are underdiagnosed, have high disease severity, and are significantly impacted on their HRQoL outcomes.^6^ Nearly one-third of patients with CSU in the ASSURE study reported having experienced angioedema in the past 12 months but did not have physician-reported angioedema and that mean DLQI scores were significantly higher (indicating worse HRQoL) for patients with angioedema vs. no angioedema. These patients are often overlooked, therefore increased awareness and education to promote best practice in primary and secondary healthcare is urgently required. The high numbers of angioedema cases reported in the CSU population highlights the need for CSU treatment options that also improve angioedema. In the present analysis, patients with angioedema had higher mean DLQI scores at baseline. This finding is comparable to the ASSURE-CSU study,^6^ however, the impact on patients’ dermatology-QoL was higher in the present analysis. The significant association of AAS7 score with DLQI categories clearly shows that patients that demonstrate better response on AAS7 are more likely to achieve the better dermatology-QoL outcomes, though there may also be other active confounders.

The rapid onset of action and therapeutic effects in the Phase 2b study indicate that effective treatments can improve QoL. The observation that lower rates of angioedema occurrence in patients moving from the core to the extension study warrant further studies on whether the effect of *anti*-IgE treatment on angioedema may be more sustained in comparison to its effect on urticaria in patients with CSU. It will also be interesting to explore the efficacy of anti-IgE therapy as an early intervention for patients with new onset CSU and concomitant angioedema. The CFB in urticaria activity in the present data was congruous with the CFB in AAS7 throughout the core study.

## Conclusion

Overall, the best outcomes on angioedema activity were significantly associated with complete response on patients’ disease activity status and dermatology-QoL. This emphasizes the need and the importance to achieve freedom from signs and symptoms of urticaria and treating the disease until it is completely controlled. Together with the increased disease severity reported in patients with CSU and angioedema, these findings highlight the efficacy of ligelizumab in reducing symptoms in moderate-to-severely affected patients with CSU. CSU patients with angioedema experience a higher disease activity and worse HRQoL than those without angioedema. Well tolerated and effective therapies will lead to better clinical outcomes for patients with CSU and associated angioedema.

## Abbreviations

AAS; Angioedema Activity Score, AAS7; Weekly Angioedema Activity Score, CFB; change from baseline, CSU; chronic spontaneous urticaria, DLQI; dermatology life quality index, HR; health-related, HRQoL; health related quality of life, IgE; immunoglobulin E, LS; least squares, PRO; patient reported outcome, q4w; every 4 weeks, QoL; quality of life, SD; standard deviation, SE; standard error, UAS; Urticaria Activity Score, UAS7; Weekly Urticaria Activity scores

## Acknowledgments

The authors thank Bharat Suryawanshi of IQVIA India Ltd. for statistical analysis support, Hayley Furlong, Ph.D., Gillian Brodie MSc of Novartis Ireland, and Mohammad Fahad Haroon, Ph.D. of Novartis Healthcare Pvt. Ltd. India, for providing medical writing assistance in accordance with Good Publication Practice guidelines (www.ismpp.org/gpp3).

## Funding

This work was supported by the 10.13039/100008792Novartis Pharma AG, Switzerland.

## Ethics statements

The institutional review board at each participating center approved the protocol. Patients provided written informed consent to participate in the study. The study was conducted according to the ethical principles of the Declaration of Helsinki. Data were collected by the trial investigators according to Good Clinical Practice guidelines and were analyzed by the sponsor. ClinicalTrials.gov NCT number: NCT02477332; NCT02649218.

## Author contribution

MMr, EH, AB and MMB participated in the conception and design of the study, MMz, JAB, AMG, MH, MMr, KS, WS and GS contributed to the acquisition of data, and EH and NB contributed to the analysis of the data. All authors contributed to the interpretation of the data, participated in the drafting and revisions of the manuscript and approved the final version of the manuscript.

## Authors’ consent for publication

All authors agree to publish this work. All authors confirm and agree to the editorial policy. Authors confirm that their manuscript is original, has not been published before, is not currently being considered for publication elsewhere, and has not been posted to a preprint server.

## Data availability statement

Novartis is committed to sharing with qualified external researchers, access to data and supporting clinical documents from eligible studies. These requests are reviewed and approved by an independent review panel on the basis of scientific merit. All data provided will be anonymized to respect the privacy of patients participating in the trial in line with applicable laws and regulations.

## Declaration of competing interest

**Martin Metz** reports personal fees from Amgen, personal fees from Aralez, personal fees from Argenx, personal fees from Moxie, personal fees from Novartis, personal fees from Roche, personal fees from Sanofi, personal fees from Uriach, outside the submitted work.

**Jonathan A. Bernstein** reports grants and personal fees from Novartis, Astra Zeneca, Sanofi-Regeneron, Amgen, Roche, Allakos, Celldex, CSL Behring, Takeda/Shire, Biocryst, Pharming, Ionis, Biomarin and Genentech outside the submitted work.

**Ana M Giménez-Arnau** reports roles as a medical advisor for Uriach Pharma, Sanofi and Genentech, Novartis, FAES, GSK, AMGEN, Thermo Fisher, Almirall and has research grants supported by Uriach Pharma, Novartis, and Instituto Carlos III- FEDER; she also participates in educational activities for Uriach Pharma, Novartis, Genentech, Menarini, LEO- PHARMA, GSK, MSD, Almirall, AVENE and Sanofi.

**Michihiro Hide** has received lecture and/or consultation fees from Kaken Pharmaceutical, Kyowa Kirin, Mitsubishi Tanabe Pharma, MSD, Novartis, Sanofi, TAIHO Pharmaceutical, and Teikoku Seiyaku.

**Marcus Maurer** is or recently was a speaker and/or advisor for and/or has received research funding from Amgen, Allakos, Aralez, AstraZeneca, Celldex, FAES, Genentech, GI Innovation, Kyowa Kirin, Leo Pharma, Menarini, Novartis, Moxie, MSD, Roche, Sanofi, Third Harmonic, UCB, and Uriach.

**Karl Sitz** has received research grants from AstraZeneca, Bellus, BioCryst, CSL Behring, Evidera, GlaxoSmithKline, Novartis Teva, KalVista, and has provided consultancy to BioCryst Pharmaceuticals Inc.

**Weily Soong** has been an advisor and/or clinical investigator and/or received speaker's honoraria and/or received consulting fee and/or grants and/or participated as a clinical investigator for/from the following companies: AbbVie, Aimmune Therapeutics, Amgen, AstraZeneca, Genentech, GlaxoSmithKline, Novartis, Pfizer, Regeneron, Sanofi and Teva.

**Gordon Sussman** has received research support from Aimmune, Amgen, Astra Zeneca, DBV technologies, Genentech, Kedrion S.p.A, Leo Pharma Inc., Novartis, Nuvo Pharmaceuticals Inc., Sanofi, Stallergenes, Merck, Schering Plough, Regeneron and ALK; is a medical advisor and/or has received payment for lectures from Merck, Novartis, CSL Behring, Pfizer, Anaphylaxis Canada, the Allergy Asthma and Immunology Society of Ontario and the Canadian Hereditary Angioedema Network.

**Eva Hua** is an employee of Novartis Institutes for Biomedical Research Co. Ltd., China.

**Avantika Barve** is an employee of Novartis Pharmaceuticals Corporation, East Hanover, New Jersey, United States.

**Maria-Magdalena Balp**, **Nathalie Barbier** and **Thomas Severin** are employees of Novartis Pharma AG, Basel, Switzerland.

## References

[1] Fricke J., Ávila G., Keller T. (2020). Prevalence of chronic urticaria in children and adults across the globe: systematic review with meta-analysis. Allergy.

[2] Maurer M., Weller K., Bindslev-Jensen C. (2011). Unmet clinical needs in chronic spontaneous urticaria. A GA(2)LEN task force report. Allergy.

[3] Kanani A., Betschel S.D., Warrington R. (2018). Urticaria and angioedema. Allergy Asthma Clin Immunol.

[4] Zuberbier T., Aberer W., Asero R. (2018). The EAACI/GA(2)LEN/EDF/WAO guideline for the definition, classification, diagnosis and management of urticaria. Allergy.

[5] Rye Rasmussen E.H., Bindslev-Jensen C., Bygum A. (2012). Angioedema--assessment and treatment. Tidsskr Nor Laegeforen.

[6] Sussman G., Abuzakouk M., Bérard F. (2018). Angioedema in chronic spontaneous urticaria is underdiagnosed and has a substantial impact: analyses from ASSURE-CSU. Allergy.

[7] van der Valk P.G., Moret G., Kiemeney L.A. (2002). The natural history of chronic urticaria and angioedema in patients visiting a tertiary referral centre. Br J Dermatol.

[8] Sánchez-Borges M., Caballero-Fonseca F., Capriles-Hulett A., González-Aveledo L., Maurer M. (2017). Factors linked to disease severity and time to remission in patients with chronic spontaneous urticaria. J Eur Acad Dermatol Venereol.

[9] Weller K., Groffik A., Magerl M. (2012). Development and construct validation of the angioedema quality of life questionnaire. Allergy.

[10] Staubach P., Metz M., Chapman-Rothe N. (2016). Effect of omalizumab on angioedema in H1 -antihistamine-resistant chronic spontaneous urticaria patients: results from X-ACT, a randomized controlled trial. Allergy.

[11] Maurer M., Aberer W., Agondi R. (2020). Definition, aims, and implementation of GA(2)LEN/HAEi angioedema centers of reference and excellence. Allergy.

[12] Weller K., Maurer M., Grattan C. (2015). ASSURE-CSU: a real-world study of burden of disease in patients with symptomatic chronic spontaneous urticaria. Clin Transl Allergy.

[13] Weldon D.R. (2006). Quality of life in patients with urticaria. Allergy Asthma Proc.

[14] Curto-Barredo L., Spertino J., Figueras-Nart I. (2018). Omalizumab updosing allows disease activity control in patients with refractory chronic spontaneous urticaria. Br J Dermatol.

[15] Zhao Z.T., Ji C.M., Yu W.J. (2016). Omalizumab for the treatment of chronic spontaneous urticaria: a meta-analysis of randomized clinical trials. J Allergy Clin Immunol.

[16] Maurer M., Kaplan A., Rosén K. (2018). The XTEND-CIU study: long-term use of omalizumab in chronic idiopathic urticaria. J Allergy Clin Immunol.

[17] Kaplan A., Ledford D., Ashby M. (2013). Omalizumab in patients with symptomatic chronic idiopathic/spontaneous urticaria despite standard combination therapy. J Allergy Clin Immunol.

[18] Maurer M., Giménez-Arnau A.M., Sussman G. (2019). Ligelizumab for chronic spontaneous urticaria. N Engl J Med.

[19] Arm J.P., Bottoli I., Skerjanec A. (2014). Pharmacokinetics, pharmacodynamics and safety of QGE031 (ligelizumab), a novel high-affinity anti-IgE antibody, in atopic subjects. Clin Exp Allergy.

[20] Maurer M., Rosén K., Hsieh H.J. (2013). Omalizumab for the treatment of chronic idiopathic or spontaneous urticaria. N Engl J Med.

[21] Saini S.S., Bindslev-Jensen C., Maurer M. (2015). Efficacy and safety of omalizumab in patients with chronic idiopathic/spontaneous urticaria who remain symptomatic on H1 antihistamines: a randomized, placebo-controlled study. J Invest Dermatol.

[22] Casale T.B., Bernstein J.A., Maurer M. (2015). Similar efficacy with omalizumab in chronic idiopathic/spontaneous urticaria despite different background therapy. J Allergy Clin Immunol Pract.

[23] Maurer M., Giménez-Arnau A., Bernstein J.A. (2022). Sustained safety and efficacy of ligelizumab in patients with chronic spontaneous urticaria: a one-year extension study. Allergy.

[24] Hongbo Y., Thomas C.L., Harrison M.A., Salek M.S., Finlay A.Y. (2005). Translating the science of quality of life into practice: what do dermatology life quality index scores mean?. J Invest Dermatol.

