# Supplementary material for: Ligelizumab improves angioedema, disease severity and quality-of-life in patients with chronic spontaneous urticaria
Source: World Allergy Organ J. 2022 Nov 15;15(11):100716. doi: 10.1016/j.waojou.2022.100716 (PMC9672946; doi:10.1016/j.waojou.2022.100716)
Supplement: Multimedia component 1 [file mmc1.pdf]

Supplementary Figure S1. Study design of the core Phase 2b and extension ligelizumab studies in patients with CSU inadequately controlled with H_1-antihistamines

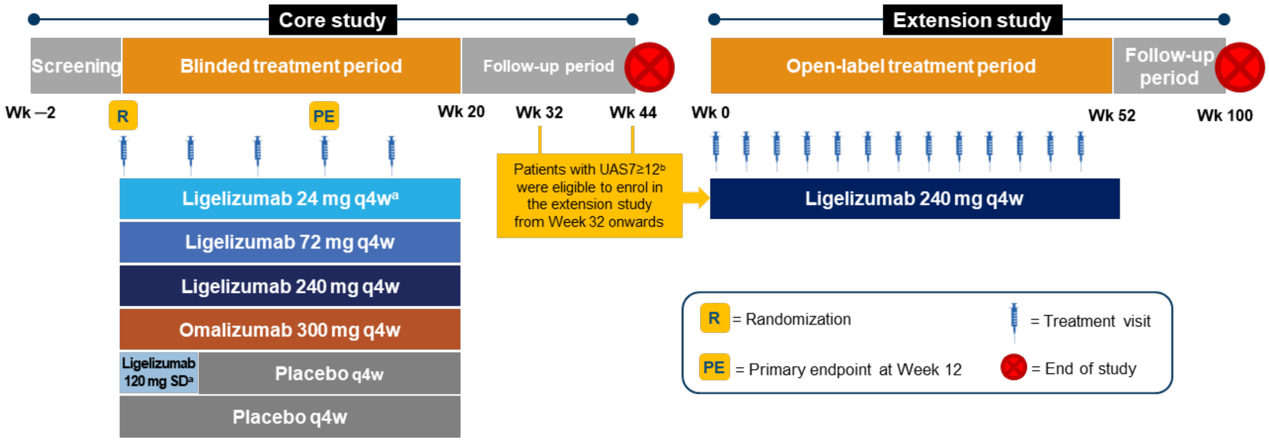

Figure adapted from Giménez-Arnau et al. Clin Transl Allergy. 2022;12(2):e12121.

[a]The ligelizumab 24 and 120 mg SD arms are not presented further as they were not relevant to outcomes presented in this analysis; [b]Patients who remained in the follow-up period for at least 12 weeks and had active disease (UAS7 ≥ 12), could enter the extension study from Week 32 onwards. CSU, chronic spontaneous urticaria; number of patients; q4w, every 4 weeks; PE, primary endpoint; R, randomization; SD, single dose; UAS7, weekly Urticaria Activity Score; Wk, week.

Supplementary Figure S2. Patient disposition during the Phase 2b core and extension studies

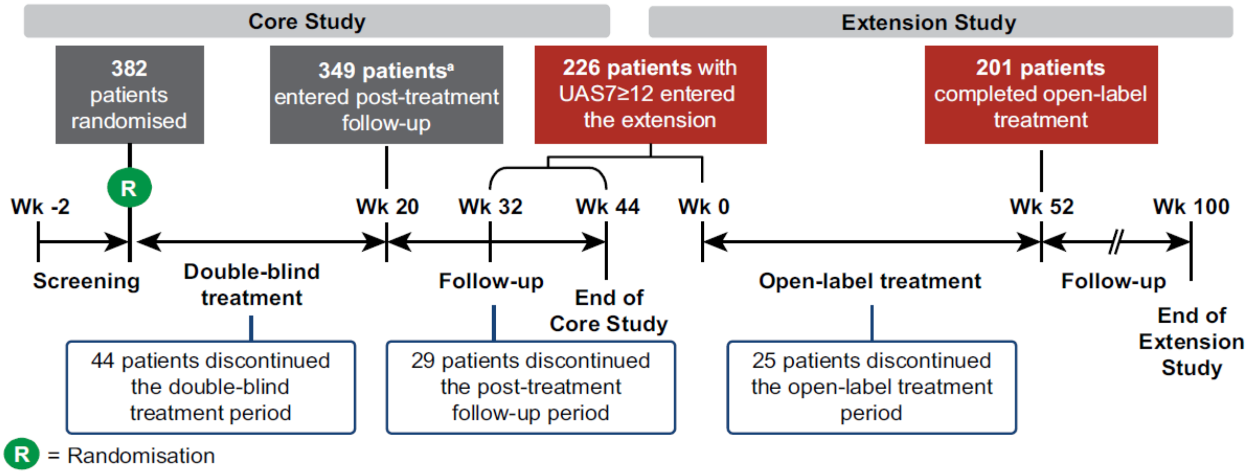

Presented at GA2LEN UCARE 2019: https://www.urticariaknowledgecenter.novartis.com/GUF_UCARE/presentations/UCARE%202019/UCARE%202019_Ligelizumab%20Reduces%20Rescue_Poster.pdf. UAS7, weekly Urticaria Activity Score; Wk, week. [a]Patients who discontinued treatment during the double-blind period were encouraged to remain in the study for the safety analysis and enter the post-treatment follow-up
